# Experimental Investigation of the Mechanical Properties of the Sand–Concrete Pile Interface Considering Roughness and Relative Density

**DOI:** 10.3390/ma15134480

**Published:** 2022-06-25

**Authors:** Chen Chen, Qi Yang, Wuming Leng, Junli Dong, Fang Xu, Limin Wei, Bo Ruan

**Affiliations:** 1 School of Civil Engineering, Central South University, Changsha 410075, China; chenchen_1203@csu.edu.cn (C.C.); wmleng@csu.edu.cn (W.L.); dongjunli@csu.edu.cn (J.D.); fangxu@csu.edu.cn (F.X.); lmwei@csu.edu.cn (L.W.); ruanbo@csu.edu.cn (B.R.); 2 MOE Key Laboratory of Engineering Structure of Heavy Haul Railway, Central South University, Changsha 410075, China; 3 Huan Tieyuan Civil Engineering Testing Co., Ltd., Changsha 410075, China

**Keywords:** interface direct shear test, sand–concrete pile interface, roughness, relative density, mechanical properties

## Abstract

The aim of this study was to investigate the effect of roughness and relative density on the mechanical properties of sand–concrete pile interface. A series of direct shear tests were carried out on the interface using a large-scale direct shear apparatus with various relative densities of sand (73%, 47%, and 23%) and concrete blocks with four roughness values (*I* = 0, 10, 20, and 30 mm). Various mechanical properties (such as shear stress, volume change, peak shear strength, secant friction angle, and normalized friction coefficient) from the interface tests were compared with trends obtained from the pure sand direct shear test. For the smooth interface, the shear stress–horizontal displacement curves of the dense sand specimen exhibited a slight softening response, which became more apparent as the roughness increased. The curves of the loose sand specimen demonstrated a hardening response. The volumetric response was influenced by the combination of normal stress, relative density, and roughness. The peak shear strength demonstrated a nonlinear increasing trend as the normal stress increased. With an increase in the normal stress, the secant friction angle and peak friction coefficient decreased as exponential and power functions, respectively. Additionally, a critical roughness value *I*_cr_ resulted from the tests, which halted the upward trend of the peak friction coefficient and normalized the secant friction angle when *I* exceeded *I*_cr_.

## 1. Introduction

Soil–structure interface interaction problems widely exist in practical engineeringsuch as retaining walls, soil nailing, soil–geotextiles interfaces, and soil–bored pile interfaces [1,2,3,4,5,6]. Soil–structure interaction is achieved mainly through load transfer at contact surfaces. Therefore, understanding the mechanical properties of the soil–structure interface is the basis for solving soil–structure interface interaction problems. Mechanical challenges involving nonlinearity, large deformation, and local discontinuity are typical complex and active topics in geotechnical engineering. Thus, research on the mechanical properties of the soil–structure interface has both theoretical significance and engineering application value.

Many studies have used test methods (direct and ring shear tests) and numerical simulation (e.g., discrete element method (DEM)) to investigate the influence of factors such as roughness, material hardness, relative compactness, particle size distribution, roundness, moisture content, and normal stress on the mechanical properties of the soil–structure interface [6,7,8,9,10,11]. Interface roughness is a critical factor affecting interface shear strength and has been extensively studied. Interface roughness is classified into “random” and “structure” types. For random roughness, Uesugi et al. [12] conducted direct shear and single shear tests on the sand–concrete interface. The author found that the relative roughness of the contact surface had a critical value, and the friction coefficient of the contact surface did not always increase linearly with the relative roughness. When the roughness was greater than the critical value, the friction factor was close to the friction coefficient of pure sand. Frost and Han [13] used the direct shearing instrument and modified interfacial shearing equipment to study the interfacial shear behavior of sand–fiber polymer. They found that the surface roughness, normal stress, initial relative density, and particle morphology of the structure had a more critical effect on shear strength. In contrast, the specimen shear rate and thickness had a more negligible impact. Dove and Frost et al. [14] conducted shear tests at the interface of Ottawa sand and a glass beads–polyethylene geomembrane, and explored the effects of particle morphology and material hardness on the shear mechanism. The study was based on contact mechanics and friction theory. The results showed that a combined coupling existed between the material surface roughness and hardness, which jointly affected the interface mechanical properties. On soft surfaces under a high normal stress, the contribution of the “plough-slip”, produced by particle morphology, to shear strength cannot be ignored. Han et al. [15] performed an interface direct shear test to study the influence of surface roughness, particle geometry, and aggregation on the interface friction angle between sand and steel with different corrosion degrees. For “structural” roughness, Hryciw et al. [16] conducted direct shear tests on the interface between sand and ribbed steel plates. They found that optimal rib spacing not only prevents the particles from blocking the grooves during shearing, but also fully mobilizes the soil to participate in the deformation coordination to form “passive resistance”. Chen et al. [17] proposed an improved sand-pouring method based on the morphological characteristics of regular roughness to measure interface roughness, and conducted an interface shear test of the red clay–concrete interface using a large-scale direct shear apparatus. The results showed that the roughness had a significant effect on the interfacial shear strength, and the interface peak strength increased with increasing roughness. Su and Zhou et al. [18] used an improved direct shear instrument to study the effect of roughness and soil particles on the interface shear strength between sand and a toothed steel plate. The results showed that the effect of interface roughness on the interface shear strength was greater than that of particle aggregation.

Field tests’ results have shown that soil relative density has a significant impact on soil–structure frictional resistance, especially for structures such as cast-in-place piles and diaphragm walls [19,20,21]. Some studies have examined the effect of relative density on the mechanical properties of soil–structure interfaces. Fakharian et al. [22] used the direct shear method to study the shear behavior of the sand–rough steel plate interface under different relative densities. The author found that the initial density, normal stress, and constant standard stiffness significantly affected the interface frictional resistance and shear displacement at failure. Additionally, the relationship between the sliding friction and shear failure stages and the shear stress–tangential displacement curve under different compactness levels was quantitatively divided. O’Rourke et al. [23] used a large-scale direct shear apparatus to study the effect of relative density on the properties of the polymer–sand interface. Wang et al. [24] performed a series of large-scale monotonic direct shear and cyclic direct shear tests to study the hoop shear characteristics of the grid–sand interface under different densities. The results showed that shear softening occurred at the geosynthetic–dense sand interface, and an increase in relative density could cause a corresponding increase in the shear strength of the reinforcement–soil interface and enhance the shear dilatation. Although many studies have examined the effect of interface roughness on the shear behavior of the soil–structure interface, the concave–convex depth of the structures designed in the studies is smaller (0.001–10 mm) than that of the structural roughness in actual engineering. Moreover, it is difficult to characterize and simulate the structure surface roughness in things such as cast-in-bored piles and underground diaphragm walls. The effect of relative density on the mechanical properties of the soil–structure interface is yet to be studied systematically and comprehensively. Furthermore, few studies have reported the effects of concrete surface roughness and relative density on the mechanical properties of the sand–concrete interface.

According to the distribution probability of the in situ bored pile foundation protruding size, smooth and rough concrete blocks were constructed with surface roughness to simulate the actual surface roughness of the pile side using an improved sand-pouring method. A series of sand–concrete interface tests with different relative densities and roughness were performed using a large-scale direct shear apparatus. Furthermore, this study systematically analyzed the influence of roughness and relative density on the relationship between the shear stress and horizontal displacement, the volume response, the peak shear strength, the secant friction angle, and the peak interface friction coefficient. The results are crucial for gaining a thorough understanding of the mechanical properties of the sand–concrete interface and for developing a constitutive model of the soil–structure interface.

## 2. Roughness Design and Evaluation

The concrete block required for the direct shear test of the sand–concrete interface was constructed according to the surface roughness of the measured concrete pile, and can be used for simulating the soil–pile interface roughness in the actual projects. The precast concrete pile was cast indoors using formwork, and the pile structural surface was regarded as a smooth surface despite its slight unevenness. The concrete in situ bored piles were cast by mechanical drilling, and the pile side surface was concave and convex, which can be regarded as a rough surface.

**Figure 1:**
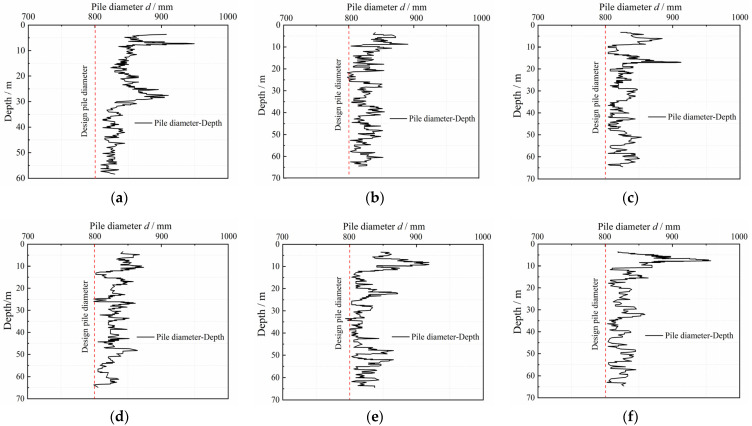
Curves of bore diameter and depth. Pile aperture curve of: (**a**) No. 3 pile; (**b**) No. 37 pile; (**c**) No. 65 pile; (**d**) No. 91 pile; (**e**) No. 207 pile; (**f**) No. 209 pile.

**Figure 2:**
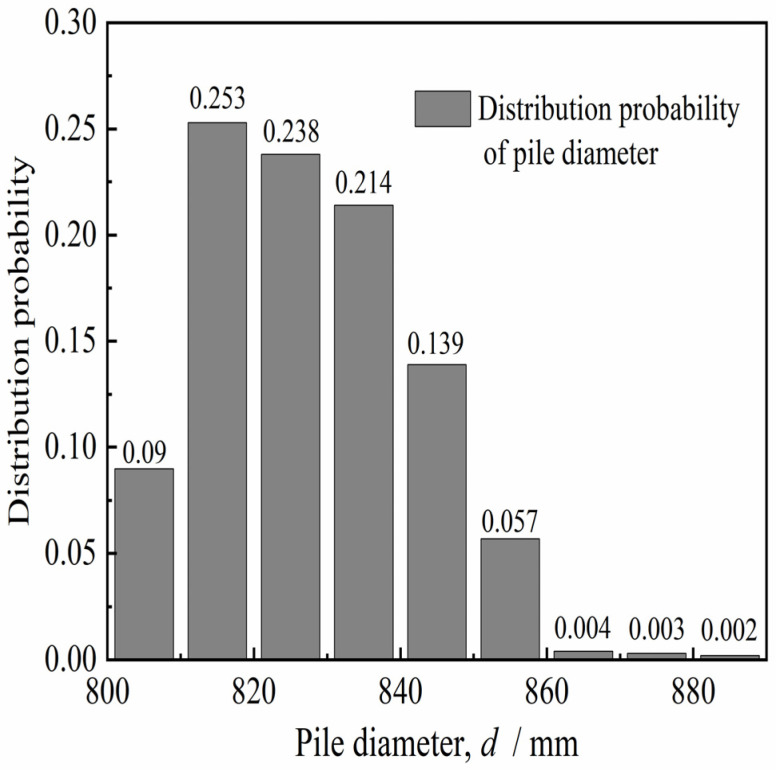
Distribution probability of pile diameter *d*.

**Figure 3:**
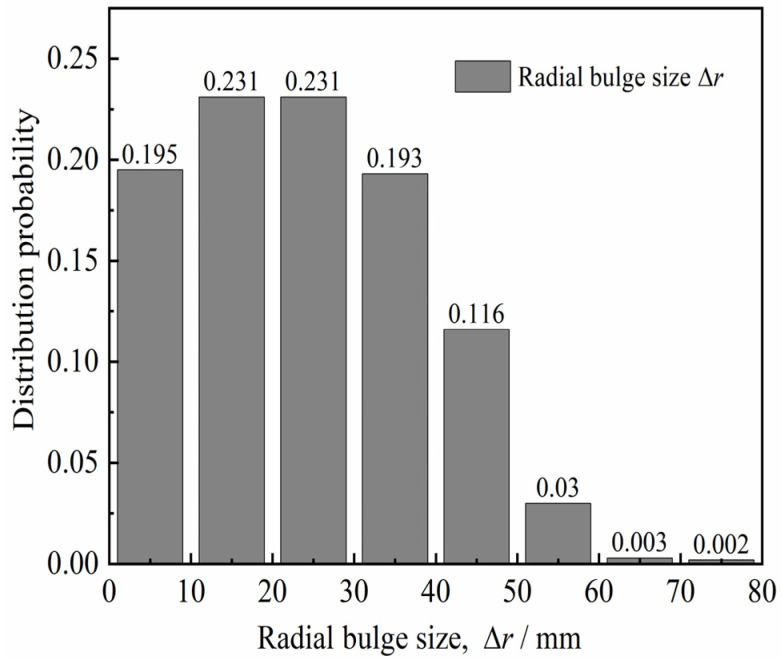
Distribution of radial bulge size Δ*r*.

We aimed to quantitatively analyze the diameter of the in situ bored pile and the variation of the concave and convex surfaces of the pile side according to the diameter detection curve of six in situ bored piles (design aperture of 800 mm) in the Pudong area of Shanghai, China [25,26]. Hence, the aperture curves were plotted as shown in Figure 1. The distribution probability of pile diameter *d* and radial bulge size Δ*r* of each pile diameter curve were obtained statistically, as shown in Figure 2 and Figure 3 (Δ*r = d/2* − *r*_min_, *r*_min_ is the minimum pile diameter after hole formation). Figure 2 and Figure 3 show that the pile diameters of the in situ bored piles were mostly in the range of 800–850 mm, and the frequency was 93.4%. The distribution frequency of the radial bulge dimension Δ*r* was as follows: the distribution frequency of 0–10, 10–20, 20–30, 30–40, 40–50, and 50–80 mm was 19.5%, 23.1%, 23.1%, 19.3%, 11.6%, and 3.5%, respectively. This phenomenon shows that the distribution frequency of the radial bulge size Δ*r* was mainly in the range of 0–50 mm, and the frequency was 96.6%; the maximum value of the radial protrusion dimension Δ*r*_max_ was approximately 80 mm. The average value $\bar{\Delta r}$ of the radial protrusion dimension was 27.6 mm.

**Figure 4:**
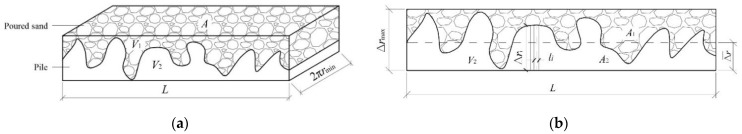
Schematic of surface roughness of pile side: (**a**) pile side cube; and (**b**) rectangular section.

The average height of the sand-pouring method represents the pile side roughness *I*. The relationship between the roughness *I* and the average value of the radial bulge dimension $\bar{\Delta r}$ was deduced as follows: expand the pile side part between the minimum and maximum pile diameters of the in situ bored pile; intercept the cube as the representative surface; divide the pile into *n* equal parts along the pile length *L* direction; and replace the surface trapezoid area with a rectangular area, as shown in Figure 4.

According to the geometric relationship illustrated in Figure 4, the sand-pouring surface area is A=2πrminL and the sand-pouring cross-sectional area is $A_{1}=V_{1}/2\pi r_{\min}$, where *V*_1_ is the volume cross-sectional area of the in situ bored pile, and *r*_max_ and *r*_min_ are the maximum and minimum radii of the in situ bored pile cross-sectional area, respectively. The average sizes of radial protrusions $\bar{\Delta r}=\sum_{i=1}^{n}\Delta r_{i}/n$, l_i_ and Δ*r_i_* are the unit length and size of the radial protruding part under the unit length, respectively. The cube height can be defined by Δ*r*_max_ = *r*_max_ − *r*_min_. As specified by the sand-pouring method, the pile side roughness can be expressed as:

(1) $$I=V_{1}/A=(V-V_{2})/A$$

Substitute the expression of each physical quantity into Equation (1) to derive:

(2) $$I=r_{\max}-r_{\min}-\bar{\Delta r}$$

**Figure 5:**
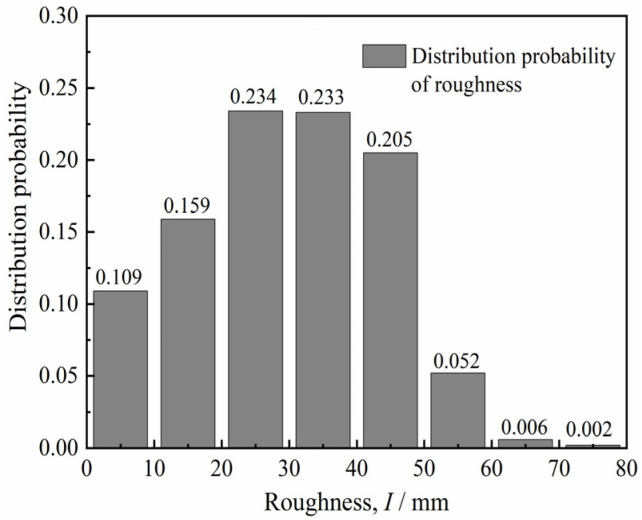
Distribution probability of pile side roughness.

The surface roughness *I* of the pile side is calculated by Equation (2). The distribution probability of the roughness *I* is obtained statistically, as illustrated in Figure 5. The distribution probability of roughness *I* in the ranges of 0–50 mm and 50–80 mm was 94.0% and 6.0%, respectively. Therefore, the roughness *I* with a higher distribution probability was taken as 10, 20, and 30 mm and as the roughness value of the test concrete block.

## 3. Experiments

### 3.1. Test Apparatus

**Figure 6:**
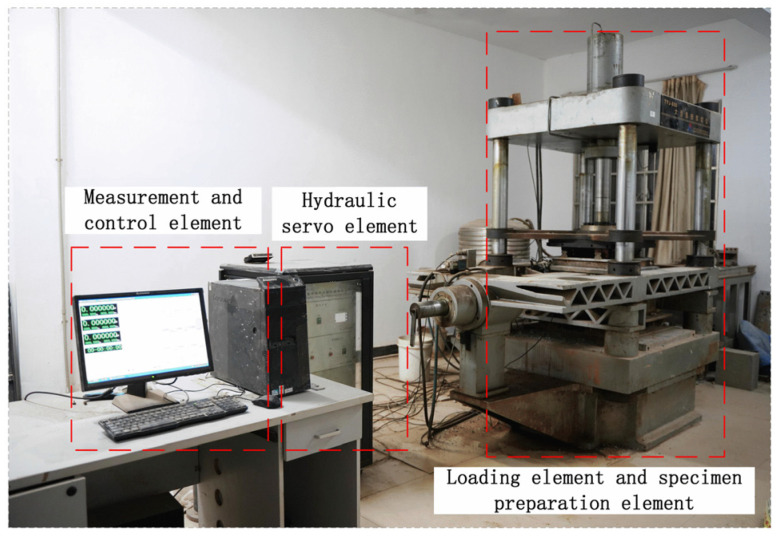
Schematic of large-scale shear test apparatus.

**Figure 7:**
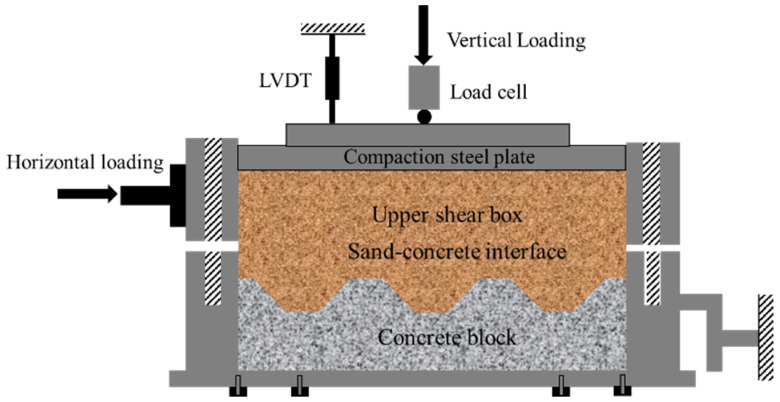
Schematic of a cross-section of the sand–concrete interface.

This study used the TYJ-800 large-scale direct shear apparatus, located in the MOE Key Laboratory of Engineering Structures of Heavy Haul Railway. As shown in Figure 6, the device consists of four parts: hydraulic servo, measurement and control, loading, and test production units. The device uses an all-digital closed-loop control system, which can automatically collect data. The length, width, and height of the upper and lower shear boxes are 500 mm, 500 mm, and 150 mm, respectively. As illustrated in Figure 7, during the interfacial direct shear test, the lower shear box is replaced with a structure and placed on the structure lift plate, composed of steel plates, screws, and nuts, and the structure with the lower shear box height is adjusted to fit the structure with the upper shear box. The length of the structure along the shearing direction is made longer than that of the upper shear box to maintain a constant shear plane during shearing and reduce the size effect of the shear box on the experimental results. The maximum shear displacement in the horizontal direction can reach 50 mm, and the shear rate control ranges from 0.0003 to 20 mm/min. Linear variable differential transformer (LVDT) sensors measure both horizontal and vertical displacements. The vertical load is applied by the hydraulic jack and reaction force frame, and the force is transmitted to the soil through the rigid plate of the sampling method, as illustrated in Figure 7.

### 3.2. Test Materials

#### 3.2.1. Soil Materials

**Figure 8:**
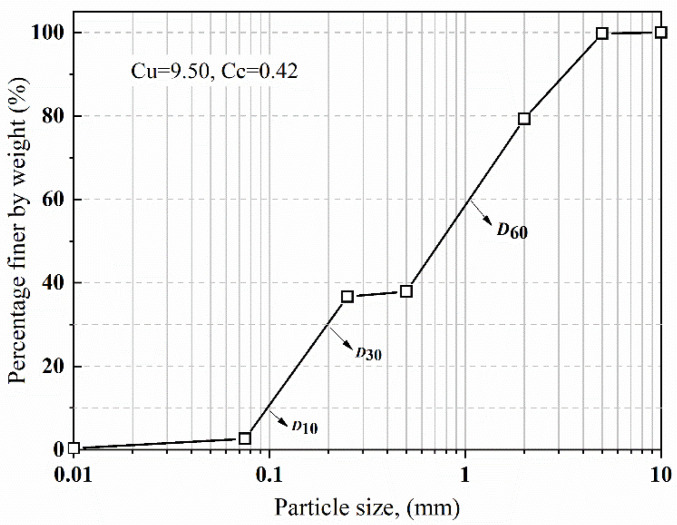
Particle size distribution of Xiangjiang River sand.

The soil used in the test was mainly derived from sand from the Xiangjiang River, located in the Hunan province of China, due to its relatively uniform grain size distribution and good control for soil relative density during compaction. Figure 8 illustrates the grain size distribution curve of the soil particle size. The sand was regarded as coarse according to the particle analysis and ASTM D2487 (ASTM 2020) [27]. The average grain size *D*_50_, maximum void ratio *e*_max_, minimum void ratio *e*_min_, and specific gravity *G*_s_ of the sand were 0.75, 0.73, 0.43, and 2.55, respectively. Table 1 presents the basic physical parameters and mechanical properties of the sand.

To explore the effect of relative density on the mechanical properties of the sand–concrete interface, three states of dense, medium-dense, and loose were set, corresponding to the three relative densities *D_r_* of 73%, 47%, and 23%, respectively. To confirm the sand sample relative densities in the test, we derived the relationship between the pouring sand sample mass *m*_s_, relative density *D_r_*, and sample volume *v* as follows. The natural void ratio *e*_0_ can be expressed as:

(3) $$e_{0}=v_{v}-v_{s}/v_{s}$$

It can be obtained as:

(4) $$m_{s}=v\cdot \rho_{s}/(1+e_{0})$$

where *v_v_* is the pore volume in the specimen, *v_s_* represents the sand particle volume in the specimen, *v* denotes the specimen volume, and *ρ*_s_ stands for the specific gravity of the sand specimen.

From the equation of relative density $D_{r}=(e_{\max}-e_{0})/(e_{\max}-e_{\min})$, the natural void ratio *e*_0_ is defined as:

(5) $$e_{0}=e_{\max}-D_{r}(e_{\max}-e_{\min})$$

where *e*_max_ and *e*_min_ are the maximum and minimum void ratios of the test sand, respectively. From Equations (3)–(5), the relationship between the pouring sand samples mass *m*_s_, relative density *D_r_*, and sample volume *v* is given as:

(6) $$m_{s}=2.55v/(1.73-0.3D_{r})$$

The initial relative density *D_r_* of the sand specimens was 73%, 47%, and 23% with a corresponding void ratio of 0.51, 0.59, and 0.66, respectively. The filling mass of the specimen was calculated by the size of the groove attached onto the concrete blocks and the upper shear box volume.

#### 3.2.2. Concrete Block

**Figure 9:**
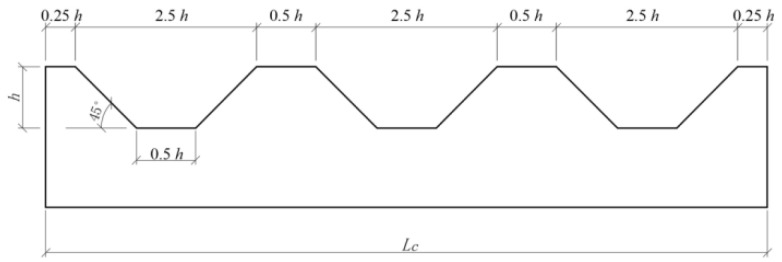
Roughness design of concrete block with grooves.

In a clay–steel interface test, Taha et al. [28] used a steel plate fabricated from milled steel rods as the lower shear box to simulate the interaction between a steel pipe pile and soil. Similarly, a smooth concrete block without grooves was fabricated to simulate the concrete precast pile surface. Meanwhile, concrete blocks with different levels of trapezoidal grooves were used to simulate the rough surface of bored piles, as illustrated in Figure 8. From the geometric relationship of the concrete block section in Figure 9, the concrete block length is obtained from the trapezoidal groove height *h*, and the sand filling volume of the concrete block is *V*_c_ = 4.5 *h*^2^*d*_c_, where *d*_c_ is the width of the concrete block.

From Equation (1), the relationship between the pile side roughness *I* and groove depth *h* on the concrete block surface can be expressed as:

(7) $$I=\frac{1}{2}h$$

**Figure 10:**
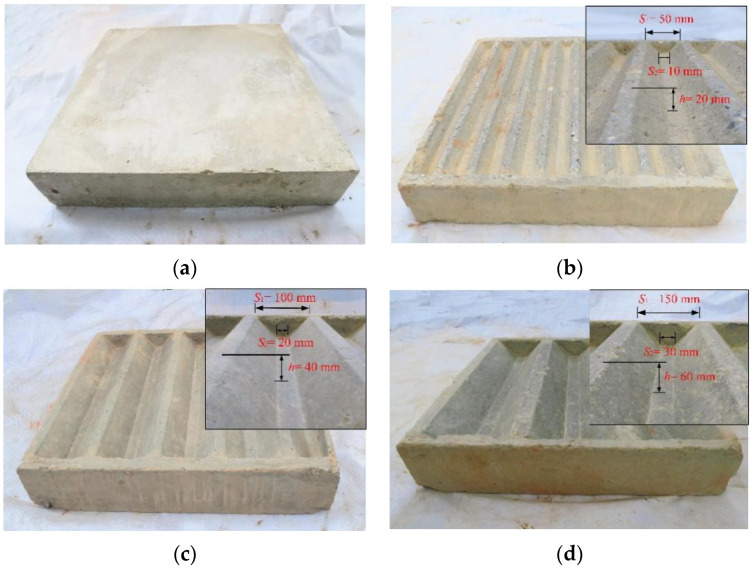
Concrete block with different roughness levels. Smooth interface (**a**); rough interface: (**b**) *I* = 10 mm; (**c**) *I* = 20 mm; and (**d**) *I* = 30 mm.

According to the pile side roughness determined by the distribution frequency of the pile side roughness and Equation (7), the trapezoidal groove height on the concrete block surface was set to 20, 40, and 60 mm. C50 concrete was used to construct concrete blocks with a length, width, and height of 570, 570, and 100 mm, respectively, as shown in Figure 10. Table 2 summarizes the parameter index of concrete material used in the tests and the measuring of the mechanical properties of the concrete blocks.

### 3.3. Test Program

The concrete block was placed on the lifting platform of the large-scale direct shear apparatus before the upper shear box was installed. The lifting platform height was adjusted to place the edge of the concrete block close to the lower edge of the upper shear box. According to Equation (6), the specimen filling mass was calculated using the preset relative density, the size of the concrete block grooves, and the upper shear box volume. The sand specimen was manually compacted into three layers in the upper shear box; according to filling height, a rammer was used to compact the sand layer-by-layer to a specified height to realize the target density. The design length of the in situ bored pile was generally less than 60 m. For consistency with actual engineering projects, the normal stress of the test loading was 50, 150, 250, and 350 kPa. The normal stress was applied with a test shear rate of 1.0 mm/min until the horizontal displacement *u* exceeded 50 mm, which satisfies the requirements of ASTM D5321 (ASTM 2012) [29]. To compare with the test results of the sand–concrete interface, the pure sand direct shear tests were carried out using the same large-scale direct shear apparatus. Table 3 details the experimental scheme.

## 4. Results and Analyses

### 4.1. Shear Stress–Horizontal Displacemenet Relationship

**Figure 11:**
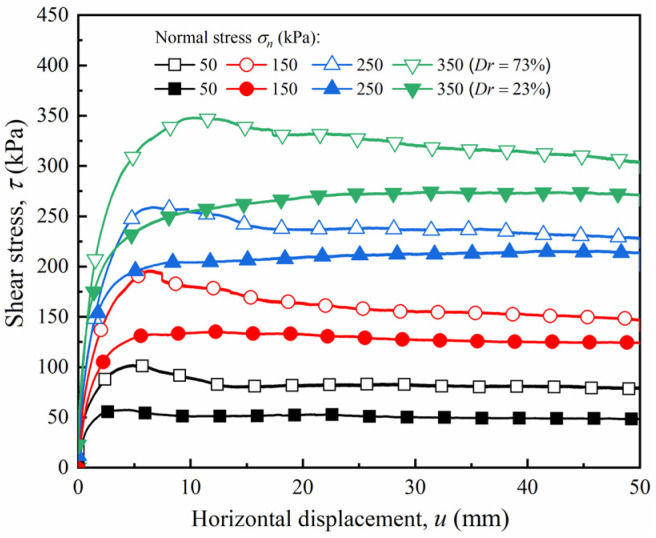
Shear stress–horizontal displacement (*τ* − *u*) curves for pure sand with different relative densities under various normal stresses.

The test results of the pure sand with different relative densities were obtained through large-scale direct shear tests. Figure 11 shows the shear stress–horizontal displacement curves of dense and loose sand, respectively. The shear stress–horizontal displacement curves of the dense sand exhibited a softening response. The shear stress increased with an increase in horizontal displacement, gradually decaying into stable residual shear stress after peaking. An increase in the normal stress on the pure sand increased the shear stress and corresponded to the increasing horizontal displacement. For example, at the normal stress ranging from 50 to 350 kPa, the peak stress strength values of dense sand were 87.2, 196.32, 259.24, and 348.36 kPa, respectively, and the corresponding horizontal displacements were 4.82, 6.42, 6.59, and 10.34 mm, respectively. In contrast, the shear stress–horizontal displacement curve of loose sand demonstrated a hardening response. With an increase in horizontal displacement, the shear stress increased rapidly at the initial stage and remained stable as the growth rate of the shear stress reduced to zero.

**Figure 12:**
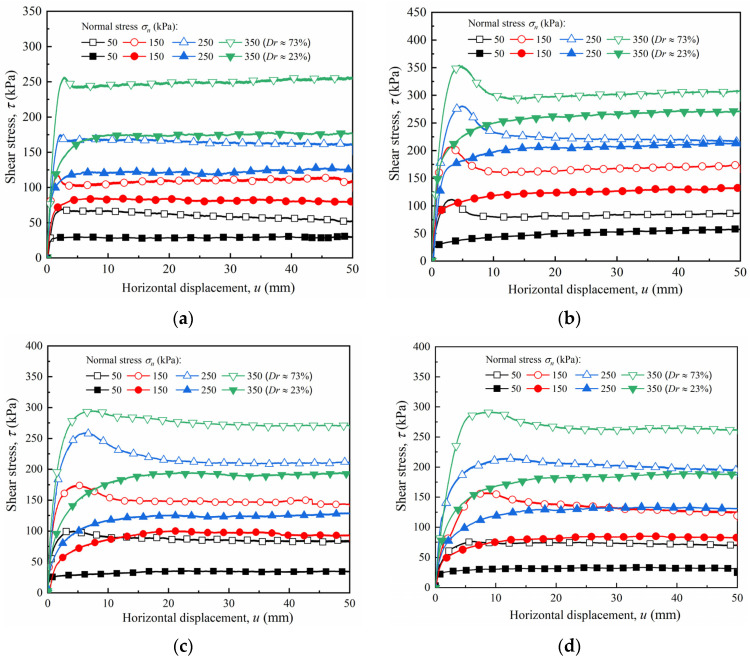
Shear stress–horizontal displacement curves (*τ* − *u*) of sand–concrete interface with different relative densities under various normal stresses: (**a**) *I* = 0 mm; (**b**) *I* = 10 mm; (**c**) *I* = 20 mm; and (**d**) *I* = 30 mm.

Figure 12 shows the shear stress–horizontal displacement curves for dense and loose sand under normal stresses ranging from 50 to 350 kPa. Figure 12a shows that, for a smooth interface, the shear stress of the dense sand increased linearly with an increase in horizontal displacement at the initial stage before slightly attenuating after reaching the peak value, which demonstrates a slight softening response. The peak strengths were 83.64, 166.32, 280.96, and 388.04 kPa, and the decay rates of the residual strength were 19.25%, 20.90%, 19.14%, and 5.93%, respectively, under normal stresses of 50, 150, 250, and 350 kPa. As can be seen from Figure 12b–d, with an increase in roughness, the shear stress–tangential displacement curves of dense sand demonstrate a prominent softening response. After the shear stress peaked, an apparent post-peak decay occurred, and the shear stress finally tended to a steady state. Additionally, an increase in roughness caused a decrease in decay amplitude after the shear stress peaked; i.e., the interface softening characteristics weakened with an increase in roughness. For instance, when the roughness *I* = 30 mm, the peak shear strengths were 76.44, 157.24, 214.92, and 264.52 kPa under normal stresses ranging from 50 to 350 kPa, respectively, and the decay rates of the residual strengths were 8.79%, 11.77%, 9.32%, and 3.51%, respectively. Compared with the smooth interface, the attenuation rate of the rough interface decreased by 10.46%, 9.13%, 9.82%, and 2.42%, respectively, which is consistent with the conclusions of Cheng et al. [30]. This phenomenon shows that, for the dense sand specimen, the surface roughness of the sand had a greater influence on the degree of development of the shear stress–horizontal displacement curve.

At the same rough interface with a dense sand specimen, the horizontal displacement corresponding to the peak shear strength increased with increasing normal stress. For example, when the roughness *I* = 20 mm, the peak strengths of the interface under the normal stress ranging from 50 to 350 kPa were 100.36, 174.44, 259.80, and 295.12 kPa, and the corresponding horizontal displacements were 4.28, 4.88, 6.41, and 7.87 mm, respectively. At the same normal stress with a dense sand specimen, the horizontal displacement corresponding to the peak strength increased with an increase in roughness. For example, when the roughness increased from 0 to 30 mm, the horizontal displacement corresponding to the peak strength at normal stresses ranging from 50 to 350 kPa increased from 2.56, 1.77, 2.17, and 2.81 mm to 6.28, 8.42, 12.65, and 15.60 mm, respectively. This phenomenon was mainly due to the increase in the roughness of the sand–concrete interface, which caused an increase in the contact area between the soil particles and the structural surface, as well as an increase in the range of soil that participated in the deformation coordination during shearing. Moreover, the horizontal displacement required to reach the failure state also increased.

For the loose sand, the shear stress–horizontal displacement curves exhibited a hardening response. As the horizontal displacement increased, the shear stress increased nonlinearly at the initial stage, and then maintained a steady state. This phenomenon shows that for loose sand, roughness had little influence on the development form of the shear stress–horizontal displacement curve, whereas relative density played a dominant role in the development form of the curve.

### 4.2. Vertical–Horizontal Displacement Relationship

**Figure 13:**
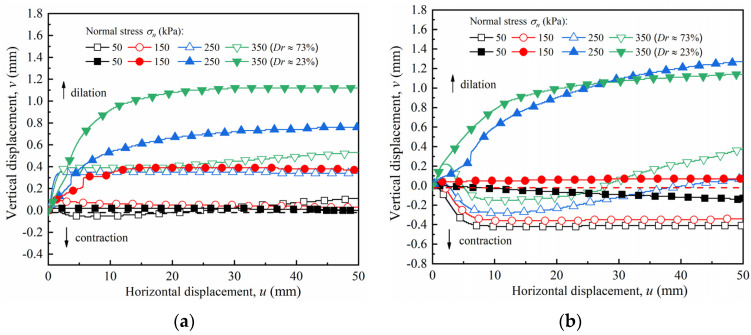

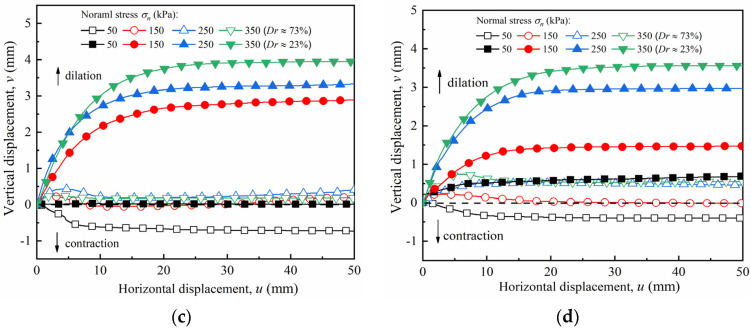
Vertical–horizontal displacement (*v* − *u*) curves of sand–concrete interface with different relative densities and normal stresses: (**a**) *I* = 0 mm; (**b**) *I* = 10 mm; (**c**) *I* = 20 mm; and (**d**) *I* = 30 mm.

Figure 13 shows the relationship curve of the vertical–horizontal displacement of the sand–concrete interface with dense and loose sand under various normal stresses. As shown in Figure 13, the volume expansion is the dilation behavior with negative values, while the volume contraction is the contraction behavior with positive values. The maximum volume change values, including those of dilation and contraction of the sand–concrete interface, are summarized in Table 4.

A slight dilation appeared in the dense sand at the smooth interface under low normal stress (50 kPa), as shown in Figure 13; the dilatancy was −0.06 mm. Under high normal stress (>150 kPa), the vertical displacement increased initially with an increase in normal stress before attaining a constant value. When the normal stress was 150, 250, and 350 kPa, the corresponding vertical displacements were 0.1, 0.51, and 0.53 mm, respectively. This result is consistent with that of Su et al. [18] and Uesugi et al. [31]. Under certain normal stresses, the friction properties of the soil–concrete interface were the main factors that determined the shear strength. There were two typical friction types: (1) the sliding friction between the particles and the contact surface; and (2) the apparent dislocation and tumbling among the adjacent particles, resulting in occlusal friction due to disengagement. The sliding friction apparently occurred at the smooth interface because of the slight occlusion between the sand particles and the interface surface during shearing, resulting in negligible volume changes. As the normal stress increased, the soil particles became constrained, and shear contraction intensified.

The increase in roughness had a considerable influence on the volume changes of the sand–concrete interface, as illustrated in Figure 13b–d. When the normal stress was below 150 kPa, the volume change of the rough interface (*I* = 10, 20, 30 mm) exhibited dilatancy. Initially, the volume change exhibited marginal contractions and then steady dilatancy as the shear stress reached a residual state. When normal stress was above 150 kPa, the volume change of the interface with *I* = 10 mm developed from contraction to dilation during the slight horizontal displacement. As the horizontal displacement intensified, the dilation phenomenon peaked and then gradually decreased. The curve rose gently and then tended toward contraction. Here, the maximum dilatation and contraction occurred simultaneously, and the values under the normal stresses of 250 and 350 kPa were −0.28/0.08 mm and −0.15/0.38 mm, respectively. This result is consistent with that of Mostafa et al. [32], who conducted a sand–geosynthetic/carbon fiber polymer interface shear test considering the influence of particle size and roughness. In his study, the critical roughness is defined as the roughness that can cause the interface volume changing to compaction first, then shear dilation, and finally shear compaction, Luo and Yao [33] reported that, at critical interface roughness, friction interlock occurred between the sand particles and the concrete surface, causing the interface shear strength and deformation to attain their limit. The soil properties and both peak and shear expansions (the critical constraint state) were obtained during this process. However, as normal stress increased, the above phenomenon was interrupted. An increase in normal stress prevented shear dilation development on the interface, resulting in the shear dilation to shear contraction transformation. As the interface roughness increased from *I* = 10 to 30 mm, the interface volume change exhibited shear dilation under a higher normal stress (>150 kPa). However, under a certain normal stress value, high roughness increased the occurrence of greater shear contractions on the sand–concrete interface. This phenomenon occurred because, under high normal stress, an increase in roughness facilitates severe dislocation and a rearrangement of the soil particles near the contact surface.

Furthermore, the interface volume change mainly showed negligible contractions and no dilation on the interface with loose sand under low normal stress. However, as normal stress increased, the volume change exhibited a considerable shear contraction. Initially, the interface contraction intensified rapidly but gradually attained stability when the contraction growth rate decreased. The shearing of the interface showed that under high normal stress values, the soil particles moved into and filled the void of the overall specimen, thereby reducing the soil specimen height and causing interface contraction.

### 4.3. Strength Parameters of Sand–Concrete Interface

#### 4.3.1. Peak Shear Strength

**Figure 14:**
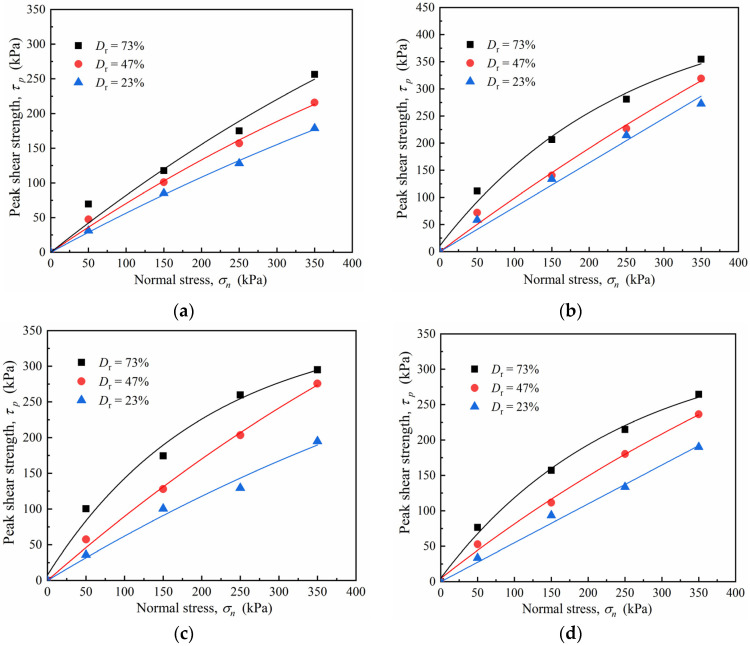
Peak shear strength–normal stress (*τ_p_* − *σ_n_*) curves of sand–concrete interface with different densities and normal stresses: (**a**) *I* = 0 mm; (**b**) *I* = 10 mm; (**c**) *I* = 20 mm; and (**d**) *I* = 30 mm.

To examine the influence of relative density and surface roughness on the shear strength of the sand–concrete interface, we considered the peak strength of the softening curves and the maximum shear stress of the hardening curves as the peak shear strength of the interface, as presented in Table 5. Figure 14 shows the peak shear strength–normal stress curves of the sand–concrete interface with different densities and normal stress values.

Figure 14 shows that, under the same roughness, the peak shear strength of the sand–concrete interface increased approximately nonlinearly with an increase in normal stress caused by a high relative density. Thus, the nonlinear increase trend of the peak shear strength of the dense sand specimen was more evident than that of the medium, dense, and loose sand. Similar results were reported by Frost and Han [13] from a direct shear test on a sand–fiber polymer interface. As shearing occurred during the sand–fiber polymer shear test, the peak and residual shear strengths increased nonlinearly with an increase in normal stress. Bishop [34] and Bolton [35] attributed this phenomenon to the dilatancy change in the soil interface. The dense soil was more likely than any other soil type to experience deformation during shearing. As the normal stress increased, the dilatancy decreased, reducing the degree of tumbling, embedding, and friction among the particles, and causing a reduction in incremental changes in the peak shear strength of the interface. Additionally, under the same relative density, the increment of the interface peak shear strength gradually decreased as the normal stress increased. For example, for the sand–concrete interface with *D_r_* = 73%, the increased peak shear strengths with roughness *I* = 10 mm were 84.57%, 36.15%, and 26.21%, while the normal stress increased from 50 to 350 kPa. This result indicates that the relative density considerably affected the shear strength relationship between the sand and concrete interface. In practice engineering, the constitutive equations should be constructed considering the actual stress conditions, the relative density, and the structure roughness [36,37,38,39].

#### 4.3.2. Secant Friction Angle

**Figure 15:**
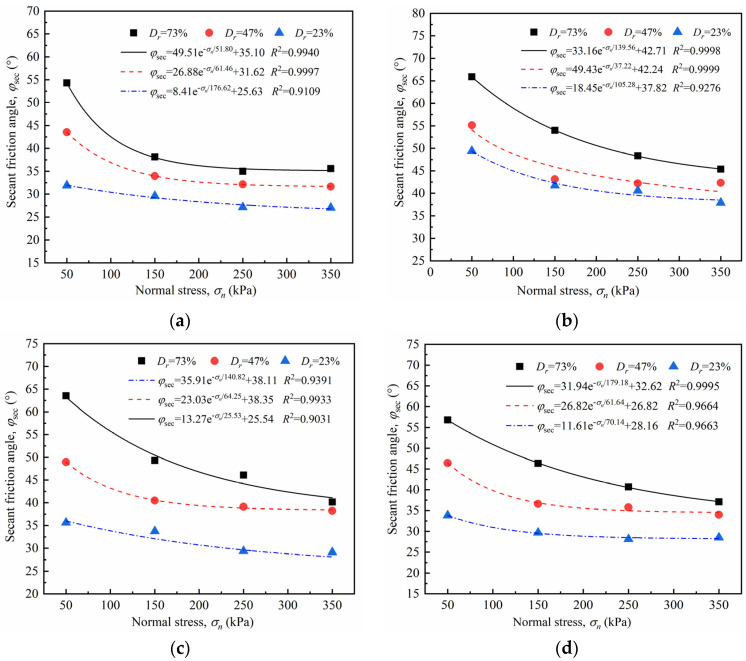
Secant friction angle–normal stress (*φ*_sec_ − *σ_n_*) curves of sand–concrete interface with different relative densities: (**a**) *I* = 0 mm; (**b**) *I* = 10 mm; (**c**) *I* = 20mm; and (**d**) *I* = 30 mm.

Owing to the nonlinearity between the peak shear strength and normal stress of the sand–concrete interface under different densities, the adoption of the secant friction angle *φ*_sec_ is typically used for characterizing the strength parameter of the interface [40,41]. Figure 15 illustrates the secant friction angle of the sand–concrete interface under different relative densities. The relationship curve of the secant friction angle and normal stress was plotted using data fitting, as shown in Figure 15. The relationship coefficient *R*^2^ of the fitting curve was above 0.9031, indicating a good correlation. The secant friction angle and normal stress can be expressed as:

(8) $$\varphi_{\sec}=Ae^{-\sigma_{n}/B}+C$$

where *φ*_sec_ is the secant friction angle of the sand–concrete interface; *σ_n_* represents the normal stress; and *A*, *B*, and *C* are the fitting parameters obtained through the data regression analysis.

Figure 15 shows that, under the same roughness levels, the interface secant friction angle reduced approximately exponentially with an increase in the normal stress; the reduction rate of the secant friction angle was initially rapid, but slowed down subsequently. For example, at *I* = 0 mm, the normal stress increased from 50 to 350 kPa, and the reduction rates corresponding to the secant friction angle of the sand specimen with *D_r_* = 43% were 22.05%, 5.27%, and 1.52%. Under the same normal stress, the higher the relative density, the larger the secant friction angle of the interface. The increasing normal stress caused a slight reduction in the secant friction angle with an increase in relative density. For the interface with *I* = 20 mm, the reduction rate of the secant friction angles corresponding to *D_r_* = 73%, 47%, and 23% was 12.93%, 2.32%, and 0.82%, respectively, under normal stress ranging from 50 to 350 kPa. This indicates that a low relative density can reduce the sensitivity of normal stress on the interface secant friction angle.

#### 4.3.3. Peak Friction Coefficient

The interface friction coefficient *μ_p_* reflects the peak shear strength *τ_p_* and interface secant friction angles *φ*_sec_, as well as the mechanical properties of the pile–soil interface. The interface peak friction coefficient *μ_p_* [41] was used to characterize the shear strength behavior of the sand–concrete interface to accurately reflect the influence of normal stress and surface roughness on the interface friction coefficient. The coefficient *μ_p_* can be defined as the ratio of the peak shear strength *τ_p_* to the applied normal stress, as follows:

(9) $$\mu_{p}=\frac{\tau_{p}}{\sigma_{n}}$$

According to Table 4 and Equation (8), the peak friction coefficients of the sand–concrete interface and pure sand under different densities were calculated, respectively; Table 5 presents the results. From the results summarized in Table 6, The curves of interface friction coefficient-normal stress of sand–concrete interface and pure sand under different densities were obtained by least square fitting, respectively, as shown in Figure 15. The fitting relationship coefficient *R*^2^ was above 0.89, indicating good correlation. The relationship between the peak friction coefficient and normal stress can be expressed as:

(10) $$\mu_{p}=A\sigma_{n}^{B}$$

where *μ_p_* is the peak friction coefficient; *σ_n_* represents normal stress; and *A* and *B* are the fitting coefficients, representing the reduction degree of the peak friction coefficient *μ_p_* with an increase in normal stress.

**Figure 16:**
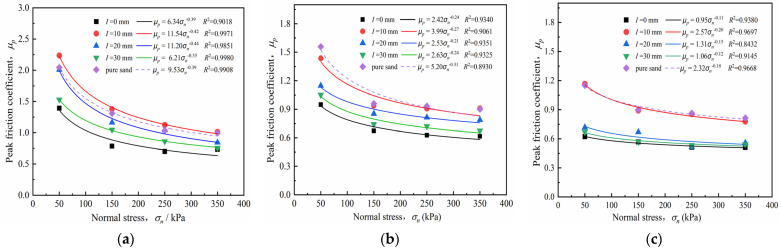
Peak friction angle–normal stress (μ*_p_* − *σ_n_*) curves of sand–concrete interface with different densities and normal stresses: (**a**) *D_r_* = 73%; (**b**) *D_r_* = 47%; and (**c**) *D_r_* = 23%.

Figure 16 shows that the peak friction coefficient reduced approximately as a power function when normal stress increased under different densities. High normal stress conditions led to a slight reduction of the peak friction coefficient. For the interface with roughness *I* = 20 mm and soil density *D_r_* = 73%, the peak friction coefficient decayed from 2.01 to 1.14 and the attenuation rate was 42.28% as the normal stress increased from 50 to 150 kPa. However, when the normal stress increased from 150 to 350 kPa, the attenuation rate of the peak friction coefficient was 8.23%. The peak friction coefficient reduced as normal stress increased; however, this phenomenon does not account for the reduction in the interface shear strength. As mentioned in Section 4.1, higher normal stress is related to higher shear stress at the same interface roughness. This indicates that, as normal stress increased, the peak shear strength changed slightly, causing a decrease in the ratio of *τ_p_*/*σ_n_*; i.e., the peak friction coefficient gradually attained stability under high normal stress. An increase in normal stress caused a simultaneous increase in the interface peak shear strength, preventing the reduction of the peak friction coefficient. This result is consistent with that of Wang et al. [42] and Zhang et al. [43], who investigated the effect of normal stress and relative density on the interface friction of a stone column–geosynthetic interface, respectively. They found that a reduction in increment of peak shear stress results in a decrease in the peak friction coefficient, rather than the peak shear strength.

Furthermore, the surface roughness had a considerable influence on the peak friction coefficient. For the interface with different relative densities, the peak friction coefficient was in the following order: $\mu_{p}^{I=10\mathrm{mm}}>\mu_{p}^{I=20\mathrm{mm}}>\mu_{p}^{I=30\mathrm{mm}}>\mu_{p}^{I=0\mathrm{mm}}$, where $\mu_{p}^{I}$ is the peak friction coefficient of the interface with a roughness value. A critical roughness *I*_cr_ = 10 mm was evident. When *I* was below *I*_cr_, the peak friction coefficient increased as roughness increased, and as *I* exceeded *I*_cr_, the peak friction coefficient reduced while roughness increased. The peak friction coefficient of the smooth interface was always the lower limit of that of the sand–concrete interface, whereas that of pure sand was not always above the upper limit. For the dense sand, the peak friction coefficient $\mu_{p}^{I=10mm}$ with critical roughness *I*_cr_ = 10 mm was greater than that of the pure sand. However, the sand–concrete interface tests for the medium and loose sand consistently produced weaker peak friction coefficients than that for the pure sand. the peak friction coefficient $\mu_{p}^{I=10mm}$ with critical roughness *I*_cr_ = 10 mm can be regarded as the upper limit among the interfaces. Similar results were reported by Irsyam et al. [16] and Jin et al. [44] after conducting shear tests on sand–plexiglass and sand–concrete interfaces, respectively. This result reflects the shear strength dynamics of the sand–concrete interface with increasing roughness.

### 4.4. Effect of Surface Roughness

The normalized secant friction angle *φ*_sec_/*φ_s_* was used to study the shear strength of the sand–concrete interface to examine the roughness effect on the mechanical properties of the interface. It was also used to determine the shear failure position during shearing, where *φ*_sec_ is the secant friction angle of the contact surface, and *φ*_s_ is the secant friction angle of the pure sand under the same relative density.

**Figure 17:**
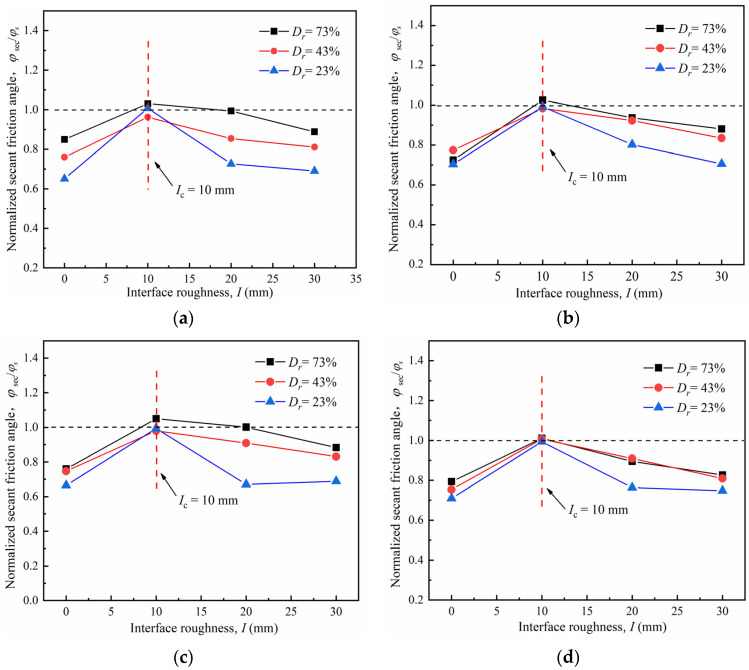
Normalized secant friction angle–interface roughness (μ*_p_* − *I*) curves of sand–concrete interface with different densities: (**a**) *σ_n_* = 50 kPa; (**b**) *σ_n_* = 150 kPa; (**c**) *σ_n_* = 250 kPa; and (**d**) *σ_n_* = 350 kPa.

Figure 17 illustrates the relationship between the secant friction angle and roughness for the sand–concrete interface under different normal stresses. It can be noted that the normalized secant friction angle increased as roughness increased from 0 to 10 mm under different densities, and then decreased as roughness increased from 10 to 30 mm. The variation of the normalized secant friction angle with different roughness values indicates the existence of critical roughness *I*_cr_; i.e., when *I < I_cr_*, *φ*_sec_/*φ_s_* increased as roughness also increased; when *I* ≥ *I_cr_*, *φ*_sec_/*φ_s_* decreased as roughness increased. As the normalized secant friction angle peaked at the critical roughness, the shear strength of the soil particles was fully mobilized and peaked. Moreover, the soil above the interface experienced ultimate deformation. These findings correspond with those of Qian et al. [45,46], who suggested the optimal thread spacing for studying the pull-out bearing capacity mechanism of grouting threaded piles. At the optimal thread spacing (identical to the critical roughness), the soil shaping area around the pile sank and the pull-out bearing capacity peaked. At the same relative density, the normalized secant friction angle *φ*_sec_/*φ_s_* satisfied the following order: $\varphi_{\sec}^{I=10mm}/\varphi_{s}$ > $\varphi_{\sec}^{I=20mm}/\varphi_{s}$ > $\varphi_{\sec}^{I=30mm}/\varphi_{s}$ > $\varphi_{\sec}^{I=0mm}/\varphi_{s}$. This conclusion is consistent with that of the peak friction coefficient as mentioned before.

The shear failure position of the sand–concrete interface is usually determined by comparing the interface and the pure sand shear strengths. Shear failure usually occurs at a position with a relatively low shear strength. For the medium dense and loose sand, the normalized secant friction angle *φ*_sec_/*φ_s_* was below 1.0; thus, the interface shear strength was lower than that of the pure sand and shear failure may occur on the sand–concrete interface. On the contrary, the *φ*_sec_/*φ_s_* for the dense sand were closer to 1.0; when critical roughness *I_cr_* was attained, *φ*_sec_/*φ_s_* exceeded 1.0, indicating that the interface shear strength was above that of the pure sand and that shear failure surface may occur in the soil.

### 4.5. Effect of Relative Density

**Figure 18:**
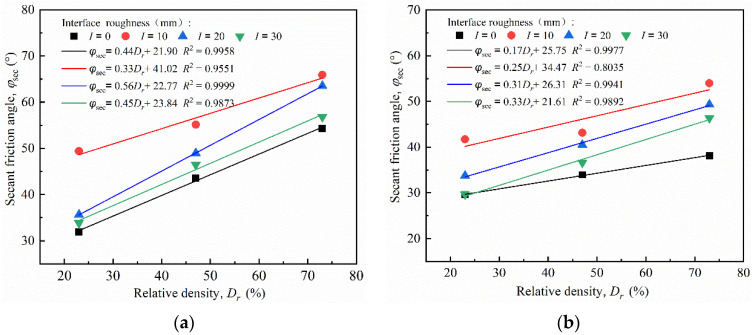

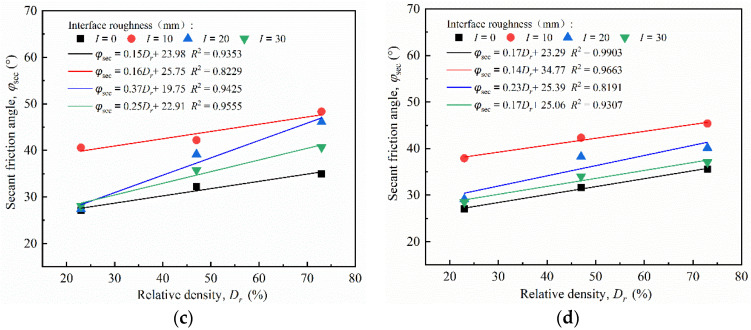
Secant friction angle–relative density (*φ*_sec_ − *D_r_*) curves of sand–concrete interface under different surface roughness: (**a**) *σ_n_* = 50 kPa; (**b**) *σ_n_* = 150 kPa; (**c**) *σ_n_* = 250 kPa; and (**d**) *σ_n_* = 350 kPa.

Figure 18 illustrates the relationship between the secant friction angle and the relative density of the sand–concrete interface under different normal stresses. From Figure 18, the secant friction angle *φ*_sec_ of the interface exhibited an increasing trend with an increase in relative density at the same normal stress. The *φ*_sec_ of the interface with *I* = 10 mm were 49.39°, 55.14°, and 65.9°, corresponding to the relative densities of 23%, 47%, and 73%, respectively, under the normal stress of 50 kPa. The secant friction angle–relative density curves were plotted using linear regression fitting, with all relationship coefficients *R*^2^ ranging from 0.8035 to 1. The fitting equation of the secant friction angle and relative density can be expressed as follows:

(11) $$\varphi_{\sec}=AD_{r}+B$$

where *A* and *B* are the fitting parameters representing the increase rate of the secant friction angle *φ*_sec_ as relative density increases as well as the influence of other factors, except relative density, on the secant friction angle *φ*_sec_. Figure 18 illustrates that the increasing rate of the *φ*_sec_ gradually reduced with the increase in normal stress *σ_n_*. For the interface with *I* = 10 mm, the increase rate of the *φ*_sec_ was 33.43%, 29.38%, 19.70%, and 19.06%, corresponding to the normal stress of 50~350 kPa. Despite the changes in relative density, the *φ*_sec_ of the smooth interface was always below that of the rough interface, whereas that of the critical roughness *I*_cr_ = 10 mm was above those of all the tests. This indicates that, unlike the smooth interface, the rough interface is required to mobilize more soil particles to facilitate shear resistance. Moreover, the effect of roughness on shear resistance does not always increase with increasing roughness.

## 5. Conclusions

In this study, concrete interfaces were constructed that could simulate actual pile side roughness using the distribution characteristics of the pile diameter curve measured in the field. A series of laboratory, large-scale direct shear tests were conducted to investigate the effects of surface roughness and soil relative density on the mechanical properties of the sand–concrete interface. The results enable a deeper understanding of the interaction of the soil–concrete interface for a regular surface roughness and various soil relative densities, and would be of benefit to practical engineering, such as in deep piles or high dams with a concrete core. The main conclusions of the study are summarized as follows:

- For the smooth interface, the shear stress–horizontal displacement curves of the dense sand exhibited a softening reaction, whereas the curves of the loose sand consistently exhibited a hardening reaction. The relative density had a dominant effect on the curve development form. In contrast, the roughness had a more considerable influence on the curve development degree.
- The smooth interface with dense sand exhibited a slight dilatancy under low normal stress, and shear contraction was apparent as normal stress increased. As roughness increased, the dense sand first contracted and then dilated under low normal stress, and the shear contraction became more evident as the normal stress increased. The loose sand retained its original shear properties despite the state of roughness.
- The peak shear strength of the interface increased nonlinearly as the normal stress increased. The greater the relative density was, the more intense the nonlinear growth became. The secant friction angle reduced exponentially as the normal stress increased, but increased linearly as the relative density increased. The peak friction coefficient *μ_p_* decreased as a power function as the normal stress increased.
- A critical roughness value *I*_cr_ = 10 mm was identified in this study. When *I* < *I_cr_*, the peak friction coefficient *μ_p_* and normalized secant friction angle *φ*_sec_/*φ*_s_ initially increased with increasing roughness *I*, but gradually decreased as *I* ≥ *I*_cr_. When *I* attained *I_cr_*, *φ*_sec_/*φ*_s_ of the interface with dense sand exceeded 1.0, and shear failure was more likely to occur in the soil.

## Figures and Tables

**Table 1:** Basic physical and mechanical properties of Xiangjiang River sand.

Property	Value
*D*_10_ (mm)	0.11
*D*_30_ (mm)	0.198
*D*_50_ (mm)	0.75
*D*_60_ (mm)	1.24
Uniformity coefficient (*C_u_*)	9.50
Coefficient of curvature (*C_c_*)	0.42
Maximum void ratio *e*_max_	0.73
Minimum void ratio *e*_min_	0.43
Specific gravity *G*_s_	2.55
Moisture content *ω* (%)	12

**Table 2:** Parameter index of concrete material used in the tests.

Constituent	Quantity: kg/m^3^	Remarks
Cement	317	Cement strength = 49.3 MPa. Cementing material strength = 37.0 MPa Water/cement ratio = 0.40 Slump value of concrete = 180 mm Maximum water glue ratio = 0.55
Fly ash	138	
Sharp sand	596	
Coarse aggregate	1210	
Water	184	
Admixture	4.60	
Density	2450	

**Table 3:** Testing programs for sand–concrete interface.

Interface Group	Groove Height *h* (mm)	Interface Roughness *I* (mm)	Relative Density *D_r_* (%)	$\mathrm{Applied}\;\mathrm{Normal}\;\mathrm{Stress}\;\sigma_{n}\;(\mathrm{kPa})$	Total Testing Specimens
I	0	0	73, 47, 23	50, 150, 250, 350	12
II	20	10	73, 47, 23	50, 150, 250, 350	12
III	40	20	73, 47, 23	50, 150, 250, 350	12
IV	60	30	73, 47, 23	50, 150, 250, 350	12
V	-	Sand–sand	73, 47, 23	50, 150, 250, 350	12

**Table 4:** Maximum volume values of sand–concrete interface (dilation and contraction).

Roughness *I*/mm	Relative Density *Dr*	Maximum Volume Change Values under Different Normal Stresses *v * (Maximum Dilation or Contraction)/mm			
		50 kPa	150 kPa	250 kPa	350 kPa
0	73%	−0.06	0.1	0.51	0.53
	23%	0.02	0.76	1.12	1.75
10	73%	−0.42	−0.36	−0.28/0.08	−0.15/0.38
	23%	−0.14	0.07	1.27	1.14
20	73%	−0.73	−0.05	0.43	0.19
	23%	0.03	2.89	3.33	3.95
30	73%	−0.4	−0.01	0.52	0.74
	23%	0.69	1.47	2.97	3.56

**Table 5:** Peak shear strength of sand–concrete interface.

Roughness *I*/mm	Relative Density *Dr*	Peak Shear Strength under Different Normal Stress Conditions *σ_n_*/kPa			
		50 kPa	150 kPa	250 kPa	350 kPa
0	73%	69.6	117.76	175	256.48
	43%	47.52	100.96	157.12	215.84
	23%	31.12	85.2	128.2	178.68
10	73%	111.8	206.36	280.96	354.6
	43%	71.8	140.52	226.68	318.88
	23%	58.32	133.76	214.24	272.52
20	73%	100.36	174.44	259.8	295.12
	43%	57.4	128	203.48	275.68
	23%	35.84	100.32	129.4	194.84
30	73%	76.44	157.24	214.92	264.52
	43%	52.6	111.44	180.2	236.24
	23%	33.52	85.44	133.56	190.04

**Table 6:** Peak friction coefficient of sand–concrete interface.

Roughness *I*/mm	Relative Density *D_r_*	Interface Peak Friction Coefficient *μ_p_*			
		50 kPa	150 kPa	250 kPa	350 kPa
0	73%	1.39	0.78	0.70	0.73
	47%	0.95	0.67	0.62	0.61
	23%	0.62	0.57	0.51	0.51
10	73%	2.23	1.37	1.12	1.01
	47%	1.43	0.93	0.90	0.91
	23%	1.17	0.89	0.85	0.78
20	73%	2.01	1.16	1.03	0.84
	47%	1.14	0.85	0.81	0.78
	23%	0.72	0.67	0.52	0.56
30	73%	1.53	1.04	0.86	0.75
	47%	1.05	0.74	0.72	0.67
	23%	0.67	0.57	0.53	0.54
sand	73%	2.04	1.31	1.04	0.99
	47%	1.56	0.96	0.93	0.90
	23%	1.15	0.90	0.86	0.81

## Data Availability

The data used to support the findings of this study are available fromthe corresponding author upon reasonable request.
